# Giant, aggressive Merkel cell carcinoma of the right arm^☆^

**DOI:** 10.1016/j.abd.2021.11.010

**Published:** 2022-12-14

**Authors:** Piotr Brzeziński, Justyna Słomka, Aleksandra Kitowska, Cesar Bimbi

**Affiliations:** a Department of Physiotherapy and Medical Emergency, Faculty of Health Sciences, Pomeranian Academy, Slupsk, Poland; b Department of Dermatology, Provincial Specialist Hospital in Slupsk, Ustka, Poland; c Private Clinic, Porto Alegre, RS, Brazil

*Dear Editor,*

In october 2018, a 65-year-old woman consults for a red, hot, painful, edematous lesion on the skin of the right arm.

Within two months of the tumor appearance, its volume increased fourfold (Fig. 1).

**Figure 1:**
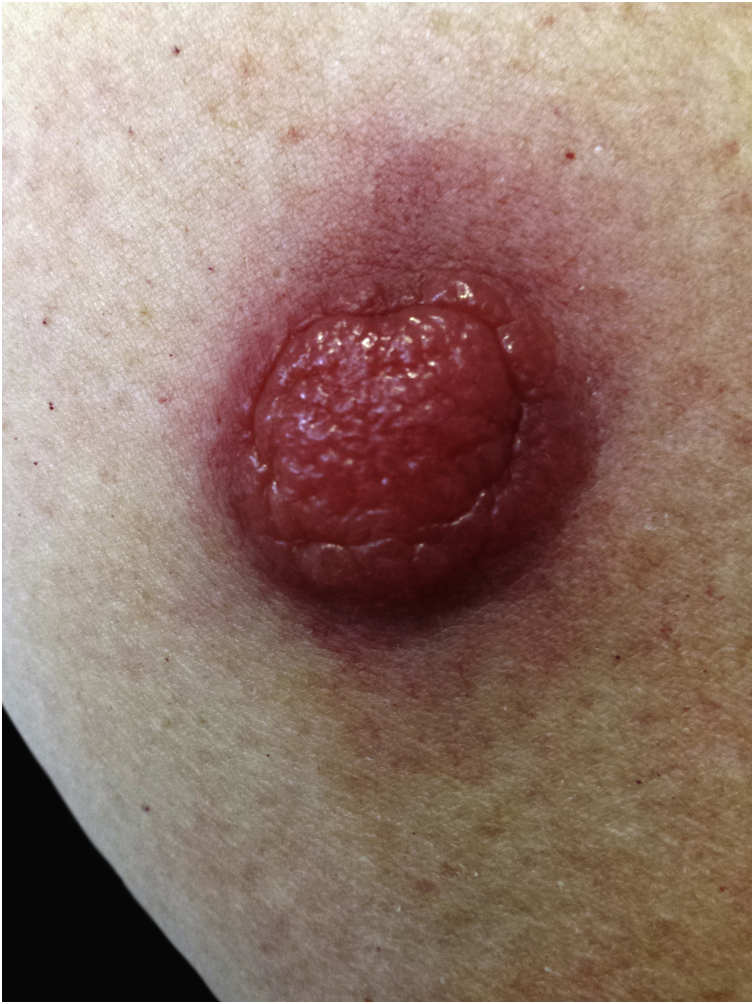
A large pink, erythematous nodule on the skin of the right arm.

The physical examination revealed an erythematous tumor 1.5 cm in diameter, movable in relation to the substrate, and poorly demarcated from the surroundings. History of comorbidities: chronic lymphocytic leukemia B-cell BCLL-PD (treated with Ofatumumab + Chlorambucil, 2008–2009).

The laboratory abnormalities showed a markedly elevated level of leukocytes: 89,600 (N: 4–10).

A 2.0 × 0.7 cm skin section with a subcutaneous tissue thickness of 0.6 cm was taken for evaluation.

In the histopathological examination of the dermis, diffuse infiltration of hyperchromatic atypical cells with a scanty cytoplasm of immunophenotype: CKAE1/3(+)"dot-like", CK20(+)"dot-like", SYN(+), CgA(±), TTF-1(−), LCA(-), Ki67(+) approx. 90%. The whole picture speaks in favor of cutaneous neuroendocrine carcinoma (Merkel cell carcinoma) (Fig. 2A and B).

**Figure 2:**
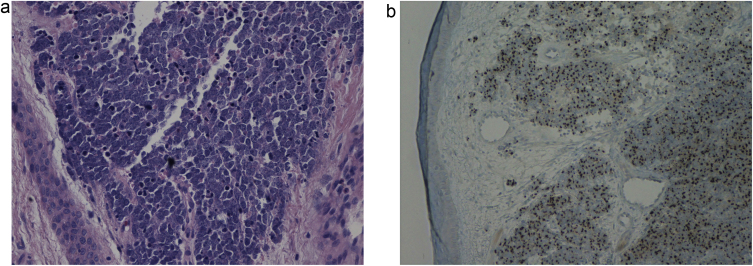
Typical histopathologic appearance of Merkel cell carcinoma. (A) Large nodular collections crowded basaloid cells in the dermis and subcutis with foci of necrosis. Aggregations of tumor cells form interanastamosing cords in the dermis in a trabecular pattern with some infiltrate strands of cells in the deep reticular dermis, (Hematoxylin & eosin, 100×). (B) CK-20 immunohistochemical staining in merkel cell carcinoma shows a paranuclear dot pattern. CK-20 40×.

The patient was transferred to the oncological surgeryward for further treatment. At the beginning of november 2018, the sentinel lymph node biopsy of the right armpit was performed together with the excision of the tumor of the right arm.

In the histopathological examination: lymph nodes with obliterated structure with infiltration of small lymphocytes of immunophenotype: CD3(−), CD20(+), CD5(+), CD23(+), cyclin D1(−), Ki67(+) 20%. The whole picture corresponds to the infiltration of small B Lymphocytes (B-SLL/CLL). In the immunohistochemical test: CKAE1/3(−), CgA(−). The histopathological exam after the excision of a skin tumor confirmed Merkell cell carcinoma.

The cycle of six chemotherapy has started (Cisplatin, Etoposide, Netupitant Palonosetron, Dexamethasone, Mannitol).

Since august 2019, aggressive disease recurred (Fig. 3A). Physical examination revealed an overgrown tumor of the right arm (35 × 16 cm) in the peak phase before radiotherapy) with a cohesive consistency, a solid arrangement of nest-like masses, surrounded by inflammation. Moreover, in the area of the right armpit and the right side, enlarged, inflammatory lymph node packages (metastases) were observed (Fig. 3B).

**Figure 3:**
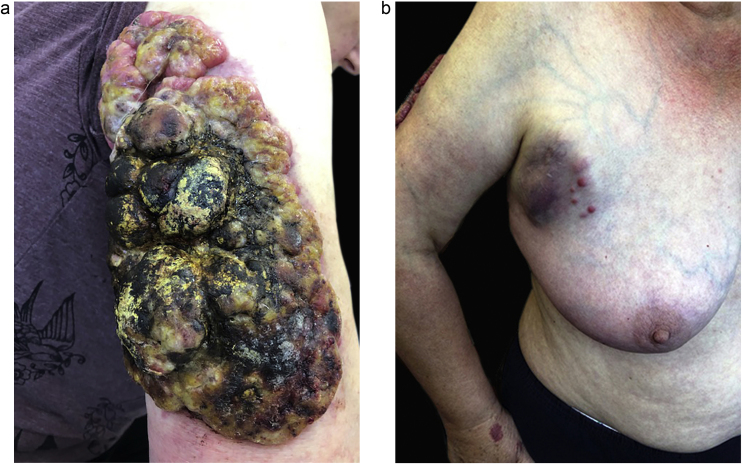
(A) Recurred of disease ‒ Giant, outgrown MCC tumor (01. September). (B) Multiple subcutaneous in transit metastases of an MCC.

Laboratory tests: lactate dehydrogenase ‒ LDH: 950, CRP: 213.

In september, started low voltage, local radiotherapy treatment of 120 Gy per fraction of 20 Gy, in 6 sessions.

After the important improvement after the second session (Fig. 4) and the surgical debridement of the tumor, there were new metastases.

**Figure 4:**
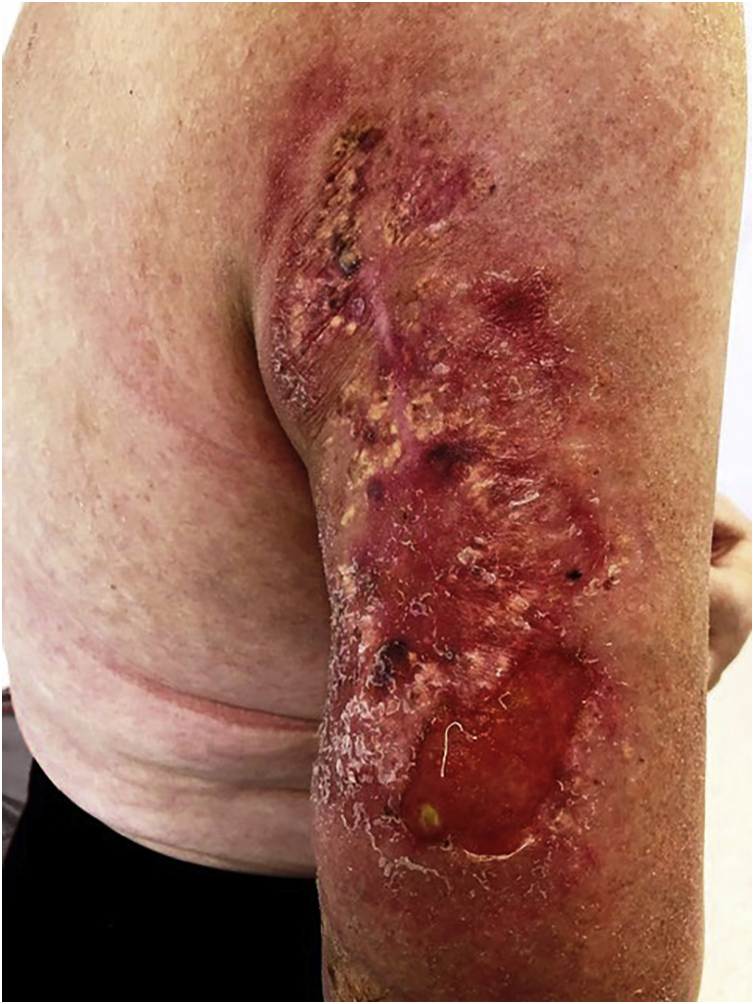
Condition after radiotherapy (first cycle) and surgical debridement.

Unfortunatley the patient died in december after 1.5 years from the Merkel cell carcinoma diagnosis.

## Discussion

Merkel cell carcinoma is an aggressive neuroendocrine carcinoma with a high rate of metastasis and 5-year overall survival^1^, ^2^.

It occurs preferentially in the elderly or immunocompromised and is most often localized in a photoexposed area, in particular on the face. The average age at diagnosis being 69 years^3^.

The discovery of a new polyomavirus, McPyV, has shed a different light on the pathogenesis of this tumor. The integration of a polyomavirus-like virus clone into the genome of Merkel tumor cells has been objectified (named “Merkel cell polyomavirus” ‒ MCPyV), thus suggesting a viral etiology. However, the viral infection alone is not enough to induce a malignant transformation^4^, ^5^.

Currently, it is still unclear whether the polyomavirus is associated with MCC or the UV is associated with MCC.

Due to the clinically unspecific primary lesion, the clinical diagnosis of Merkel cell carcinoma it is often done late. The acronym AEIOU was developed to provide assistance in finding the diagnosis: A = Asymptomatic/lack of tenderness, E = Expanding rapidly (doubling in 3 months), I = Immunosuppression, O = Older than 50 years, U = Ultraviolet-exposed skin site^6^. Our case met the five above criteria.

So far, 12 cases of giant MCC tumors have been described.

The largest tumor of the MCC was described by authors from the USA on the right forearm of an 84-year-old man (23 × 18 cm)^7^. This particular patient responded well to radiation therapy.

Authors from Italy described a giant Merkel cell carcinoma of the left arm of a 76-years-old woman (13 × 10 cm)^8^.

So far, a larger tumor than the one we presented has not been described (35 × 16 cm).

The treatment of choice for MCC is still the complete excision with a 1–2 cm safety margin and subsequent irradiation in the tumor bed regional lymph node stations^9^.

Regardless of the SLNB result, adjuvant irradiation of the primary tumor bed is recommended for all patients.

Advances in the treatment of MCC in recent years have led to the FDA-approved drug ‒ the immune checkpoint inhibitor avelumab, a PD-1 antagonist.

In the case of our patient, this drug was reported to the Polish healthcare system in order to obtain it for treatment. However, the patient did not manage to take this drug.

The course of MCC is rarely as aggressive and as fast as in our patient. Failure of chemotherapy and radiotherapy, multiple metastases, and death 1.5 years after diagnosis were certainly related to the deficiency of the immune system.

## Financial support

None declared.

## Authors' contributions

Piotr Brzeziński: Concepts; Design; Clinical studies; Data acquisition; Statistical analysis; Manuscript preparation.

Justyna Słomka: Concepts; Design; Definition of intellectual content; Clinical studies; Data analysis; Manuscript preparation; Manuscript editing; Manuscript review.

Aleksandra Kitowska: Clinical studies; Data acquisition; Data analysis; Statistical analysis.

César Bimbi: Definition of intellectual content; Clinical studies; Data analysis; Statistical analysis; Manuscript editing; Manuscript review.

## Conflicts of interest

The authors have no conflict of interest to declare.
